# Ultralow Platinum Content in Defect-Rich Tungsten Disulfide: Approaching Platinum Performance in Proton Exchange Membrane Water Electrolyzers

**DOI:** 10.1021/acsami.6c03423

**Published:** 2026-04-13

**Authors:** Elena Puentes-Prado, Esdras J. Canto-Aguilar, Alice Kuzhikandathil, Mouna Rafei, Tugce Ustunel, Eduardo Gracia-Espino

**Affiliations:** † Department of Physics, 8075 Umeå University, Umeå SE-901 87, Sweden; ‡ Permascand AB, Folketshusvägen 50, Ljungaverk SE-841 99, Sweden

**Keywords:** water electrolysis, proton exchange membrane, tungsten disulfide, platinum, hydrogen production

## Abstract

Tungsten disulfide in its metallic 1T phase is a stable and efficient electrocatalyst for the hydrogen evolution reaction. However, stabilizing the 1T phase while maintaining high conductivity and catalytic activity is challenging. Here, we addressed these issues by producing few-layered WS_2_ with an expanded interlayer distance of ∼10 Å, followed by the incorporation of foreign metals such as Ni, Co, or Pt. These dopants allow tuning of the 1T/2H phase ratio and limit the production of oxidized species such as WO_*x*_ and SO_*x*_. In particular, the addition of only 0.3 at% of Pt leads to a preferential formation (∼70%) of the 1T-WS_2_ phase. Despite the ultralow Pt content, Pt-WS_2_ exhibited a 41% reduction in the overpotential required to reach −10 mA cm^–2^, a 78% decrease in charge transfer resistance, and a 14-fold increase in active surface area compared to pristine WS_2_. The excellent catalytic activity of Pt-WS_2_ is attributed to the presence of the 1T phase with a higher density of active sites, enhanced conductivity, and stronger hydrogen interaction, all facilitated by the presence of Pt. In addition, Pt-WS_2_ shows great performance when used as a cathode in proton exchange membrane water electrolyzers, achieving a current density of 1.75 A cm^–2^ at 2.1 V, 270% larger than that of pristine WS_2_.

## Graphical abstract

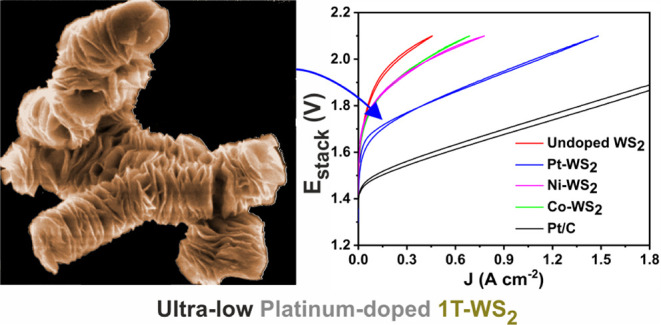

## Introduction

Expensive and scarce platinum-group metals (PGMs) remain the backbone of PEM (proton exchange membrane) water electrolysis. In particular, platinum, ruthenium, and iridium are considered the most active electrocatalysts for the hydrogen evolution reaction (HER) under acidic conditions.^1^ While promising alternative materials such as metal phosphides, metal chalcogenides, and transition metal nitrides are being explored, they currently exhibit several limitations.^2^ These include slower HER kinetics, a lower availability of catalytically active sites, reduced conductivity, and reduced operational stability. Even when these alternative materials demonstrate HER performance (e.g., Tafel slopes and overpotentials) comparable to platinum, their performance at high current densities remains consistently underwhelming.

Therefore, strategies to reduce the use of PGMs rather than eliminate them are the preferred approach. For example, transition metal dichalcogenides (TMDCs) such as MoS_2_ and WS_2_ are known as highly attractive candidates for the HER.^3–5^ But their thermodynamically preferable 2H phase displays limited HER activity due to the inert nature of the basal plane and poor electrical conductivity. However, the transition toward metallic polymorphs such as 1T or 1T′ improves their catalytic performance by activating the basal plane, increasing electrical conductivity, and facilitating charge transfer to adsorbed species.^6–8^ Additional strategies such as defect engineering,^9,10^ edge site exposure,^11^ and heteroatom doping,^6^ have shown to further improve their intrinsic catalytic performance when evaluated in a conventional three-electrode cell configuration.

In particular, the incorporation of PGMs such as Pt,^12,13^ Pd,^14,15^ or Ru^16,17^ even at trace levels can induce phase transformation, modulate the local electronic structure, stabilize reactive sites, and introduce new active centers that mimic the coordination environment of bulk PGMs. Zhong and collaborators reported that Pt-doped MoS_2_ (Pt ∼ 0.03 mg cm^–2^) achieved an overpotential to reach 10 mA cm^–2^ (η_10_) of only 46 mV.^18^ Gupta et al. obtained an η_10_ of 89 mV with Pd-doped WS_2_.^19^ Siva and Vasu reached an η_10_ of 113 mV with Ru-doped (10 at%) WS_2_.^16^ And Chae et al. achieved an η_10_ of 73 mV with 1.0 wt % Pt-doped WS_2_.^20^ The observed performance was attributed to improved charge transfer and the coexistence of the 1T and 2H crystal phases, collectively reducing kinetic barriers, improving hydrogen interaction, and enhancing long-term stability. However, the role of the metal dopants in modulating the phase composition, electronic structure, and active site distribution of TMDCs remains an active area of research. Moreover, systematic comparisons under both three-electrode and PEM water electrolyzer conditions are still limited.

Despite these advances, the vast majority of studies considering TMDC-based cathodes have been conducted exclusively under three-electrode half-cell conditions, which do not fully capture the demanding operational environment of proton exchange membrane water electrolyzers. In PEMWE devices, cathode materials are exposed to high current densities, strongly acidic conditions, and new electrode–membrane interfaces. Under these conditions, catalyst behavior can deviate significantly from that observed in half-cell measurements. Recent studies on molybdenum-based materials operating in full PEM electrolyzers have demonstrated that, while non-noble catalysts generally do not match the performance of Pt, they can operate at high current densities for extended periods under realistic conditions.^21,22^ However, systematic investigations specifically addressing WS_2_-based cathodes in full PEM electrolyzer configurations remain extremely limited, and direct correlations among phase composition, dopant effects, and device-level PEMWE performance are still lacking.

The present work aims to bridge this gap by investigating expanded multilayered WS_2_ containing a mixture of 2H/1T crystal phases as cathode material for water electrolysis. We examined the effects of ultralow incorporation of Pt, Ni, and Co on the structural, spectroscopic, and electronic properties of WS_2_, and assessed how these modifications influence electrocatalytic performance. The materials are evaluated not only under conventional three-electrode HER conditions but also in a full PEM water electrolyzer configuration, providing direct insight into the feasibility and limitations of WS_2_-based cathodes under technologically relevant operating conditions.

## Experimental Methods

### Chemical Reagents

Ammonium tetrathiotungstate ((NH_4_)_2_WS_4_), N,N-dimethylformamide (HCON­(CH_3_)_2_, 99.8%), cobalt­(II) acetylacetonate (Co­(C_5_H_7_O_2_)_2_), nickel­(II) acetylacetonate Ni­(C_5_H_7_O_2_)_2_, platinum­(II) acetylacetonate Pt­(C_5_H_7_O_2_)_2_, and sulfuric acid (H_2_SO_4_, 95–97%) were acquired from Merck. Absolute ethanol (99.96%) was purchased from VWR. Nafion solution (5 wt % in alcohol) was obtained from Ion-Power. Deionized water (DI, Milli-Q, 18.25 MΩ) was obtained through an EMD Millipore water purification system. All of the reagents were used as received.

### Material Characterization

Scanning electron microscopy (SEM) was carried out using a Carl Zeiss Merlin microscope with an energy-dispersive X-ray (EDX) spectrometer. The elemental composition was evaluated by measuring 2 different spots along the sample. The average value is reported. X-ray photoelectron spectroscopy (XPS) was performed with a Kratos Axis Ultra DLD electron spectrometer utilizing a monochromatic Al Kα (1486.6 eV) X-ray source operated at 150 W output power. The deconvolution of the peaks in the W 4f region was used to determine the relative content of 1T- and 2H-WS_2_.^11,23–25^ All binding energies were corrected by using carbon (C 1s = 284.6 eV). X-ray powder diffraction (XRD) was conducted on a PANalytical X’pert diffractometer (Cu Kα, λ = 1.5406 Å, 45 kV, 40 mA) with a scanning range of 10–80°, a step size of 0.01395°, and 0.5 s per step. The interlayer spacing associated with the (002) reflection of WS_2_ was evaluated using the Bragg’s law *d* = λ/2 sin θ, where λ corresponds to the wavelength of Cu Kα (1.5406 Å), and θ is half of the measured diffraction angle of the (002) peak. Raman spectroscopy was performed on a Renishaw Qontor Raman spectrometer using a 532 nm laser diode (10% power) calibrated to a Si crystal at 521 cm^–1^. Each spectrum consisted of 3 accumulations of 3 s. High-resolution transmission electron microscopy (HRTEM) studies were performed on a Glacios cryo-TEM instrument (Thermo Scientific) with an acceleration voltage of 200 kV. The elemental composition of the Pt-WS_2_ sample was also evaluated by X-ray fluorescence (XRF) spectroscopy. XRF is a bulk technique, and it was performed using an ARL Quant’X Energy-Dispersive Analyzer (Thermo Fisher) under ambient conditions. The instrument is equipped with a rhodium anode as the X-ray source. A collimator (8 mm) was used to define the analysis spot. For quantitative analysis, the UniQuant (Thermo-Scientific) software was employed. The system was calibrated using nine reference elements (Al, Ti, Cr, Fe, Ni, Mo, Sn, W, and Pb).

### Synthesis of WS_2_ and TM-WS_2_

The synthesis of WS_2_ and TM-WS_2_ was carried out using a hydrothermal method.^26,27^ To prepare WS_2_, 0.17 mmol of (NH_4_)_2_WS_4_ was added to 15 mL of (CH_3_(CH_2_)_7_NH_2_ adjusting the pH to 4 with H_2_SO_4_, and the mixture was sonicated for 30 min under N_2_ flow. The solution was transferred into a 30 mL Teflon-lined stainless-steel autoclave. The autoclave was sealed and heated up to 180 °C for 24 h. Afterward, the samples were filtered, washed repeatedly with distilled water and absolute ethanol, and later air-dried at 80 °C for 10 h. TM-doped WS_2_ was synthesized following the same procedure as that for undoped WS_2_, with the addition of the dopant (Pt, Co, or Ni). The concentration of the foreign metal relative to tungsten was 2.5 at% for Pt (1.67 mg of precursor, equivalent to 0.00425 mmol of Pt), 5 at% for Co (2.19 mg of precursor, 0.0085 mmol of Co), and 5 at% for Ni (2.18 mg of precursor, 0.0085 mmol of Ni). Under the current synthesis conditions, the actual doping content was reduced to 0.3 at% for Pt-WS_2_, 1.8 at% for Ni-WS_2_, and 0.9 at% for Co-WS_2_, according to EDX, XRF, and XPS studies. The samples were labeled TM-WS_2_, where TM indicates the type of dopant.

### Electrochemical Measurements

The working electrode was prepared by dispersing 5 mg of catalyst in 50 μL of a solution containing a Nafion solution (5% in alcohol), 300 μL of ethanol, and 900 μL of DI water, followed by sonication for 1 h. Afterward, 20 μL of the catalyst suspension was drop-cast onto a glassy carbon electrode (Ø = 2.5 mm) and dried under vacuum. A standard three-electrode setup was employed with a glassy carbon rotating disc (PINE, 0.196 cm^2^) as the working electrode, an Ag/AgCl (3 M KCl) as the reference electrode, and a graphite rod as the counter electrode. Data acquisition was carried out using an Ivium Technologies potentiostat. The electrolyte solution (0.5 M H_2_SO_4_, pH = 0) was degassed with N_2_ for 15 min before each measurement. A scan rate of 5 mV s^–1^ was used for cyclic voltammetry (CV) scans in the range of 0.0 to −1.0 V vs RHE. To achieve a stable polarization curve, 15 CVs were conducted, and the final cathodic scan is reported. The Tafel slopes were evaluated from an additional CV scan at 1 mV s^–1^. All potentials were referenced to the reversible hydrogen electrode (RHE) using the Nernst equation: E_RHE_ = E_cell_ + 0.059 × pH + E_Ag/AgCl_, where E_cell_ is the cell potential vs Ag/AgCl and E_Ag/AgCl_ is 0.197 V. The polarization curves were compensated for 90% internal resistance (*iR*). Electrochemical impedance spectroscopy (EIS) was performed at a fixed potential of −0.2 V vs RHE for all samples, covering a frequency range from 100 kHz to 0.1 Hz (10 mV amplitude). The data were modeled using a Randles circuit to determine the ohmic resistance (R_Ω_) and the charge transfer resistance (R_ct_). The double-layer capacitance (C_dl_) was determined by CV scans conducted at varying scan rates (5, 20, 40, 60, 80, and 100 mV s^–1^) within ± 50 mV of their respective open circuit potential. The electrochemical surface area (ECSA) was evaluated using ECSA = C_dl_/35 μF cm^–2^, where 35 μF cm^–2^ is the specific capacitance for a flat surface in acidic conditions.^24,25^

### Single-Cell PEM Water Electrolyzer Test

The activity of the WS_2_-based cathode electrocatalysts was tested as part of membrane-electrode assemblies (MEAs) in a proton exchange membrane (PEM) water electrolysis cell. The MEAs were assembled by hot-pressing the anodes and cathodes with a commercial Nafion N-115 polymer electrolyte membrane using a Stahls Hotronix 6 in. × 6 in. heat press. The Nafion membranes were previously activated at 80 °C for 1 h in H_2_O_2_ (3% v/v), deionized water (18.2 MΩ·cm), 0.5 M H_2_SO_4_, one more time in water, and stored in the same solvent until usage. The anodes were fabricated by spraying an IrO_*x*_ ink onto a platinized-Ti fiber felt (heated at 100 °C) as the gas diffusion layer (GDL). The ink was deposited by using an airgun with N_2_ as the carrier gas. The ink consisted of IrO_*x*_ nanoparticles (10.6 mg mL^–1^) dispersed in a mixture of 2-propanol, water, and a Nafion solution (5 wt % in ethanol, Nafion D520) with a ratio of 88.10:10.04:1.86 v/v. A similar procedure was used to prepare the cathodes, where the ink consisted of 5.5 mg mL^–1^ of Pt/C (20% Pt/C), or TM-WS_2_-based catalyst, in a 2-propanol/water/Nafion D520 (84.9:9.7:5.4% v/v) mixture. Carbon paper (CP-39BB, heated at 70 °C) was used as GDL for the cathodes. The catalyst loading was ∼2.6 mg cm^–2^ of IrO_*x*_ for the anodes, and ∼0.50 mg cm^–2^ of Pt or ∼0.9 mg cm^–2^ of TM-WS_2_ materials for the cathodes. An electrolyzer test station (Scribner) was used to evaluate the performance of the fabricated MEAs with a geometrical active area of 4 cm^2^, a working temperature of 80 °C, atmospheric pressure, a feed flow of 100 mL min^–1^ of water, without N_2_-flow at the cathode side. A systematic characterization protocol involving activation/relaxation steps, galvanostatic step sweep polarization curves, galvanostatic electrochemical impedance spectroscopy, and chronoamperometric procedures was used. A detailed description of the conditions and the sequence of the measurements performed is presented in the corresponding section. The long-term operational stability of the Pt-WS_2_ MEA was evaluated by applying a constant current density of 0.25 A cm^–2^ for 170 h, followed by 1.0 A cm^–2^ for 380 h.

### Computational Details

The computations were performed using density functional theory (DFT) at the GGA-RPBE level as implemented in the SIESTA code version 4.1.5.^28^ A double-ζ polarized basis was used to represent the valence electrons, and an energy cutoff of 350 Ry was used for charge and potential integration. All systems were geometrically optimized with a conjugate gradient minimization until the maximum forces were less than 0.04 eV Å^–1^. The surfaces used for the hydrogen adsorption studies were constructed as single-layer hexagonal plates containing 235 atoms for 1T-WS_2_ and 237 atoms for 2H-WS_2_. A single Pt, Ni, or Co atom was introduced as a dopant by replacing a W atom in the center of the disk. The adsorption free energy of hydrogen (ΔG_H_) was evaluated using the computational hydrogen electrode model using $\Delta G_{H*}=\Delta E_{H}+\Delta E_{\mathrm{ZPE}}-T\Delta S_{H}$ .^29–31^ ΔE_H_ is the hydrogen adsorption energy using a H_2_ molecule as a reference state. ΔE_ZPE_ is the change in zero-point energy between the adsorbed and gas phase, and ΔS_H_ is the change in vibrational entropy of the adsorbed and gas phase. It has been reported that the zero-point energy and entropy changes for the HER are equal to 0.29 eV in transition metal dichalcogenides.^32^ ΔG_H_ was evaluated at low hydrogen coverage as expected for weakly interacting materials.

## Results

Expanded multilayered WS_2_ comprising a mixture of the 2H and 1T crystal phases was produced via hydrothermal synthesis. Electron microscopy studies (SEM and HRTEM) reveal that the as-produced WS_2_ is composed of interconnected nanowires (Figure 1a, Figures S1–S2) with little to no formation of the characteristic plates expected for a layered WS_2_. The addition of transition metals as dopants changed the structure, morphology, and phase distribution. Among the three TM-WS_2_ (TM = Pt, Ni, or Co), the addition of Pt promotes the formation of thin plates agglomerated in worm-like structures (Figure 1b, Figures S1 and S3). HRTEM studies reveal an expanded basal plane with an interlayer distance of ∼10 Å. Ni-WS_2_ exhibits structures similar to those seen in Pt-WS_2_ but with thinner and smaller plates that appear to roll up in a flower-like morphology (Figure S4a). Co-WS_2_ formed irregular scales with no defined features (Figure S4b). Energy-dispersive X-ray (EDX) elemental mapping revealed a uniform distribution of all elements across the surface, with no significant agglomeration (Figure S5). However, we observed that the content of all dopants was very low. For Pt-WS_2_, EDX indicated a Pt content of 0.1 at%. X-ray photoelectron spectroscopy (XPS) revealed a Pt content of 0.3 at%. Bulk elemental analysis using X-ray fluorescence spectroscopy (XRF) showed a Pt content of <1 ppm, below the detection limit. And HRTEM studies did not show the presence of Pt nanoparticles, in agreement with the ultralow Pt loading. All of these techniques clearly indicate that the Pt content in Pt-WS_2_ is very low. For Co-WS_2_, EDX (XPS) revealed a Co content of 1.8 at% (1.0 at%), and for Ni-WS_2_, EDX (XPS) indicated a Ni content of 0.9 at% (0.6 at%). A summary is given in Table S1. Despite the low dopant content, we observed clear differences in the morphology and lamellar arrangement between pristine WS_2_ and all other metal-doped WS_2_. The presence of Pt, Co, or Ni during the synthesis induces clear structural modifications. This behavior is consistent with previous studies on 2D transition-metal dichalcogenides, where ultradilute or monatomic metal species have been shown to induce lattice perturbations and structural changes through interactions with sulfur vacancies, edges, or locally distorted regions without the formation of metallic clusters.^12,33,34^

**Figure 1:**
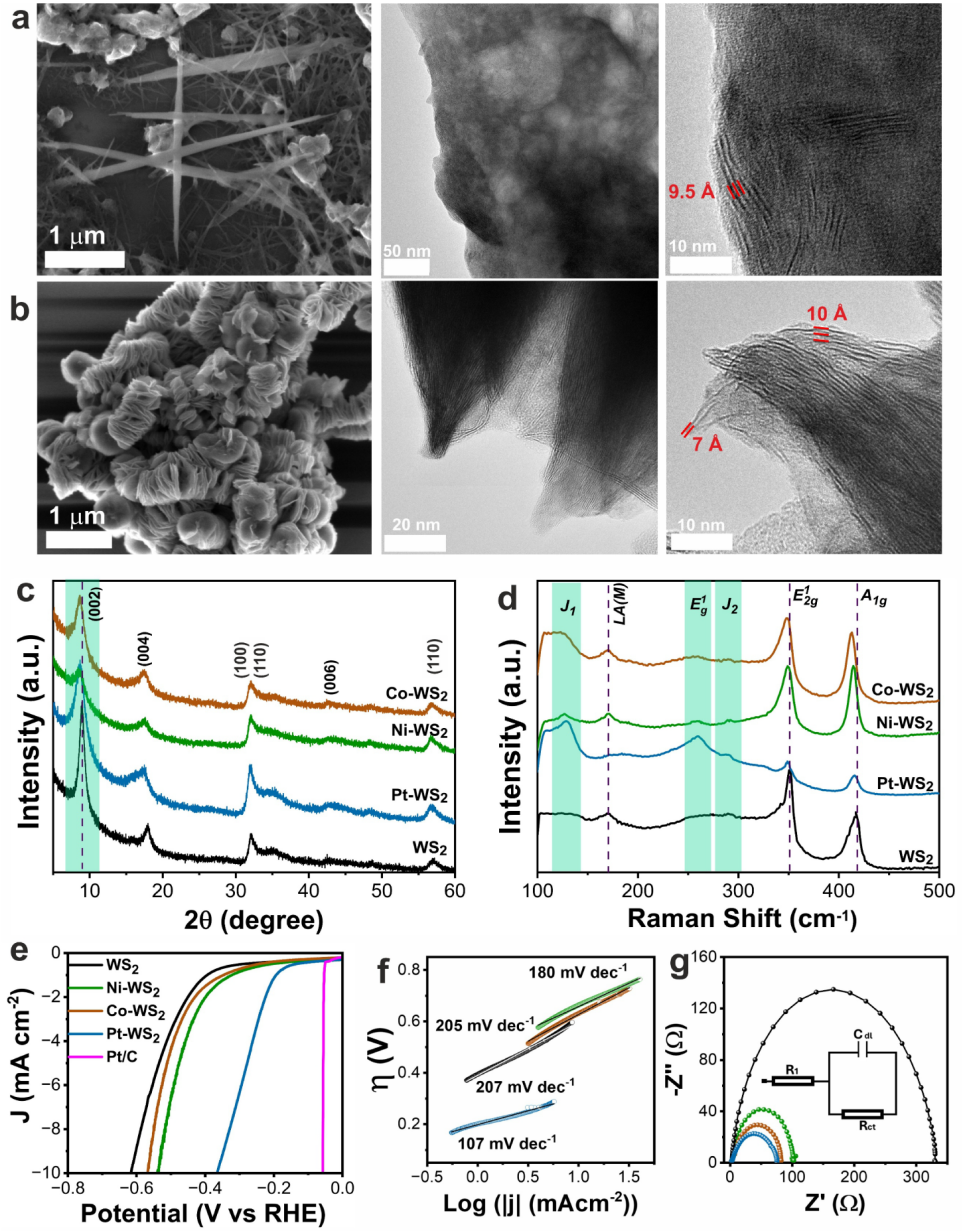
Structural characterization and electrochemical performance. SEM and HRTEM images of (a) WS_2_ and (b) Pt-WS_2_. (c) XRD patterns, (d) Raman spectra, (e) HER polarization curves (0.5 M H_2_SO_4_), (f) Tafel plots, and (g) Nyquist plots of WS_2_ and TM-WS_2_. The HER performance of commercial 20% Pt/C is included in (e) for comparison.

X-ray diffraction studies (Figure 1c) indicate the presence of layered WS_2_ with an expanded interlayer distance along the (002) plane of ∼10 Å, as compared to its ideal bulk counterpart of 6.2 Å.^35^ The interlayer distance was determined from the position of the (002) diffraction feature using the Bragg’s law. The interlayer spacings for the (002) planes of WS_2_, Pt-WS_2_, Ni-WS_2_, and Co-WS_2_ are 9.8, 10.1, 10.3, and 10.2 Å, respectively. Note that the interplanar distance is very similar irrespective of the doped element, despite differences in their ionic radii. This indicates that the expanded interlayer is neither originated nor affected by the dopants, and there must be other reasons behind the intercalation.^26,35–37^ The expanded interlayer distance was also observed by HRTEM studies (Figure 1a–b), where certain regions exhibited a wave-like characteristic with variable interlayer distances. The Raman spectra of WS_2_ exhibited characteristic features (170.7, 350.8, and 415.8 cm^–1^) corresponding to the LA­(M), E^2^_1g_, and A_1g_ modes (Figure 1d). The TM-WS_2_ also exhibited these features, but a slight red shift was observed for the E^1^_2g_ and A_1g_ bands (Table S2), likely associated with the lattice strain caused by the dopants. The LA­(M) mode was observed in all samples, except Pt-WS_2_. The absence of this signal suggests a lower defect density or a reduced degree of disorder in this material, in agreement with the fact that the LA­(M) band is typically activated by defect- or edge-related scattering and by resonance processes, rather than being a fundamental Raman mode.^38,39^ Additionally, the TM-WS_2_ exhibited the E_1g_ band (258.8 cm^–1^) and two other low-frequency bands (J_1_ and J_2_) characteristic of the metallic 1T phase.^26,40^ Finally, features corresponding to WO_3_ were observed (Figure S6) with the O–W–O stretching mode and the asymmetric stretching mode of oxygen bridges (O–W–O) seen at 691 and 802 cm^–1^, respectively.^41^

The electrocatalytic performance toward the hydrogen evolution reaction was evaluated using a standard three-electrode configuration in an acidic medium (0.5 M H_2_SO_4_, pH = 0). Figure 1e shows the *iR*-corrected polarization curves. The HER performance of a commercial 20 wt % Pt/C catalyst is included for comparison, where it exhibited an overpotential to achieve −10 mA cm^–2^ (η_10_) of 60 mV. On the other hand, undoped WS_2_ required an overpotential to achieve −10 mA cm^–2^ (η_10_) of 614 mV, consistent with previous reports.^11,42,43^ The incorporation of Pt significantly reduced the η_10_ to 360 mV (−42% compared to WS_2_). While Ni-WS_2_ and Co-WS_2_ exhibited η_10_ of 537 (−13%) and 568 (−8%) mV, respectively. Tafel analysis also reveals fast HER kinetics in Pt-WS_2_ with a Tafel slope of 107 mV dec^–1^ (Figure 1f), corresponding to the Volmer–Heyrovsky mechanism with electrochemical desorption as the rate-determining step (rds). In comparison, the Tafel slopes for Ni-WS_2_, Co-WS_2_, and undoped WS_2_ are 180, 205, and 207 mV dec^–1^, respectively. The larger value of the Tafel slopes indicates the Volmer step (hydrogen adsorption) as rds. This weak hydrogen interaction is characteristic of metal dichalcogenides,^26,30^ and in this case, Ni and Co fail to improve likely due to their low atomic concentration. Electrochemical impedance spectroscopy studies (Figure 1g) reveal that Pt-WS_2_ exhibits the lowest charge transfer resistance (R_ct_) among all of the evaluated materials, corresponding to a 78% reduction relative to undoped WS_2_ (Table S3). The trend in R_ct_ values is Pt-WS_2_ < Ni-WS_2_ < Co-WS_2_ < undoped WS_2_, see Table S3. The electrochemically active surface area (ECSA) estimated from double-layer capacitance measurements (Figure S7 and Table S3) reveals that Pt-WS_2_ exhibits an ECSA of 139.0 cm^2^ per cm^2^_geo_, which is nearly 14 times larger than that of undoped WS_2_ (9.9 cm^2^ per cm^2^_geo_). In comparison, Ni-WS_2_ and Co-WS_2_ exhibited increases to only 30.7 and 12.8 cm^2^ per cm^2^_geo_, respectively.

The catalytic activity of TM-WS_2_ was also investigated by theoretical studies using density functional theory. As expected, undoped 2H-WS_2_ exhibits an extremely weak interaction with hydrogen, seen as a large positive value of the free energy of hydrogen adsorption (ΔG_H_).^31^ In this case, the basal plane exhibits a ΔG_H_ = 1.55 eV. This agrees with the observed value of the Tafel slope in Figure 1f where hydrogen adsorption is the rds. The only sites active toward the HER are the edges of the hexagonal 2H-WS_2_ plate. This is similar to 2H-MoS_2_, where sulfur atoms at the edge have favorable adsorption energies. After the addition of dopants (Pt, Ni, or Co), the ΔG_H_ decreases indicating a stronger interaction with hydrogen, see Figure 2a and Figure S8a. This effect is only noticeable in the active sites neighboring the dopant atom, and this short-range effect has also been reported in other doped materials.^29,30,44^ In all three TM-WS_2_ systems, the activated sites exhibited an average value of ΔG_H_ = −0.2 eV, which is nearly optimal for the HER (Figure 2a and Figure S8a). But despite the excellent hydrogen interaction, most of the sites in the basal plane of the 2H-TM-WS_2_ still do not participate in the reaction.

**Figure 2:**
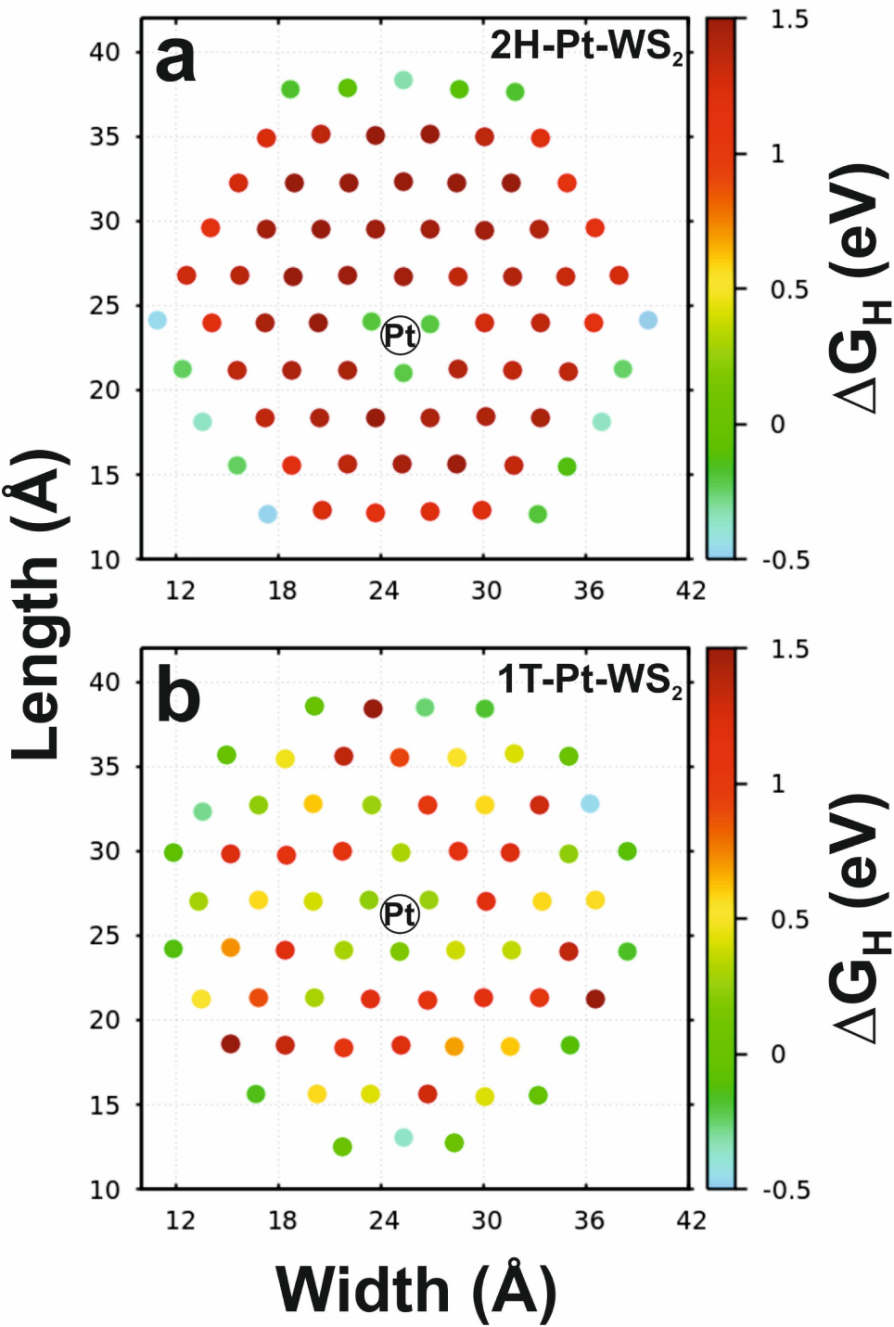
HER activity maps of Pt-WS_2_. Pt-doped WS_2_ with (a) 2H and (b) 1T phase. The position of the Pt atom is marked with a black circle. Positive (negative) values of the hydrogen adsorption free energy (ΔG_H_) indicate a weak (strong) interaction with hydrogen. Sites with optimal hydrogen interaction (ΔG_H_ ≈ 0) are green.

As seen from the theoretical studies, the basal plane of the 1T-WS_2_ is highly active toward the HER with ∼50% of the sulfur atoms having a ΔG_H_ in the range of ± 0.50 eV. This is similar to the 1T-MoS_2_, where also 50% of the basal plane is deemed active toward the HER.^26,29^ The addition of Pt, Co, or Ni into 1T-WS_2_ had a similar effect when compared to 2H-TM-WS_2_, where the enhanced HER activity was mostly limited to first neighbors reaching values of ΔG_H_ in the range of −0.15 to 0.25 eV (Figure 2b and Figure S8b). This indicates that while Pt, Ni, or Co benefits the HER activity in nearby active sites, their direct effect on the catalytic performance is minimal when the dopants are present in low concentrations. In such cases, their main role is likely to promote the formation of the 1T-WS_2_ phase, a function that only Pt achieved experimentally in the Pt-WS_2_ sample.

### Electrocatalytic Performance in a PEMWE Cell

We evaluated the performance of TM-WS_2_ as a cathode electrocatalyst in a two-electrode PEM water electrolysis cell. We used an extensive characterization protocol to evaluate the evolution of the material for more than 70 h of operation. Figure 3a depicts the test protocol applied to all of the devices. Before any experiment, a constant current of 0.01 A cm^–2^ was applied for 1 h to allow the cell temperature (80 °C) to stabilize, followed by an activation step of 0.1 A cm^–2^ for 30 min. A short stabilization step (10 min at 0.01 A cm^–2^) was applied to let the device reach steady state before a galvanostatic polarization was applied. Afterward, three galvanostatic EIS tests were carried out at 0.05, 0.125, and 0.375 A cm^–2^ (Steps I–III in Figure 3a). Further details are given in the experimental methods. To evaluate the stability of the TM-WS_2_ cathodes, two potentiostatic steps were conducted at 1.9 V for 24 h each. Additional galvanostatic curves and EIS studies were carried out after each potentiostatic study (Steps IV–VI, Figure 3a).

**Figure 3:**
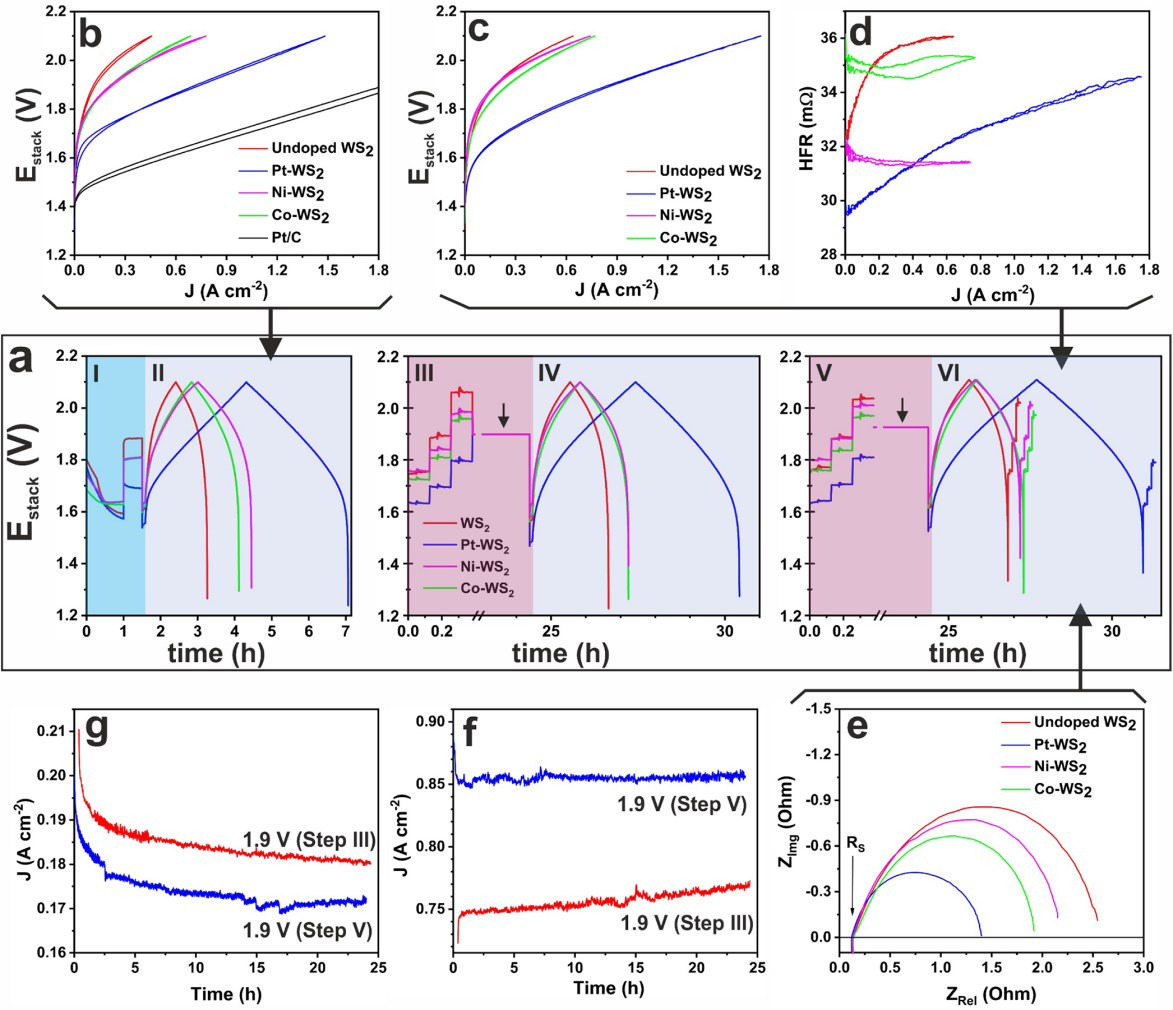
Cell performance of TM-WS_2_ cathodes in PEMWE cells. (a) Cell voltage profiles describing the characterization protocol (>70 h) for WS_2_ (red), Pt-WS_2_ (blue), Co-WS_2_ (green), and Ni-WS_2_ (magenta). (b) First and (c) last galvanostatic polarization curves. A Pt/C MEA is shown in (a) as a reference. (d) HFR as a function of current and (e) Nyquist plots corresponding to the last galvanostatic curve. Current transients for (f) Pt-WS_2_ and (g) Ni-WS_2_ of both potentiostatic studies.

Figure 3b–c shows the first and last galvanostatic polarization curves. Initially, Pt-WS_2_ exhibited the largest current density of 1.48 A cm^–2^ at 2.1 V, followed by Ni-WS_2_ (0.78 A cm^–2^@2.1 V), Co-WS_2_ (0.68 A cm^–2^@2.1 V), and WS_2_ (0.46 A cm^–2^@2.1 V). After the testing protocol depicted in Figure 3a, the maximum current density (J^Max^) achieved at 2.1 V changed. The J^Max^ values in Pt-WS_2_, Co-WS_2_, and undoped WS_2_ increased by 0.27 A cm^–2^ (+18.1%), 0.081 A cm^–2^ (+11.8%), and 0.184 A cm^–2^ (+40.3%), respectively. On the other hand, Ni-WS_2_ exhibited a slight loss in performance of −0.036 A cm^–2^ (−4.6%). The increase in performance observed in Pt-WS_2_ resulted in a current density as high as 1.75 A cm^–2^@2.1 V or 1.0 A cm^–2^@1.92 V. This current density is highly competitive among the currently existing Pt-doped TMDC electrocatalysts, with PEMWE devices achieving current densities of 1.0 A cm^–2^ at 1.82 V (10 wt % of Pt),^18^ 1.97 V (0.17 mg_Pt_ cm^–2^),^45^ and 1.71 V (2.74 wt % of Pt).^46^ For comparison, we prepared a reference MEA using commercial 20% Pt/C as the cathode (20%Pt-MEA) instead of TM-WS_2_. The polarization curves are shown in Figure 3a and Figure S9. After the testing protocol, 20%Pt-MEA achieved a current density of 1.0 A cm^–2^@1.74 V. This is just 180 mV lower than that of the Pt-WS_2_ MEA.

On the other hand, Ni-WS_2_ and Co-WS_2_ exhibited 0.78 A cm^–2^@2.1 V, while undoped WS_2_ only achieved a current density of 0.64 A cm^–2^@2.1 V. The water-splitting onset potential, calculated graphically in the kinetic-controlled regime (Figure S10), reveals the following trend: Pt-WS_2_ (1.37 V) < WS_2_ ≈ Co-WS_2_ (1.39 V) < Ni-WS_2_ (1.48 V). The trend in the onset potential does not directly reflect the trend observed for the maximum current density, indicating that other factors (e.g., different rds, electrical conductivity, and interface) might affect the performance at high current densities.

Figure 3d presents the progression of the high-frequency resistance (HFR), extracted from the polarization curves in Figure 3c, as a function of the device current density. The HFR includes all contributions of the ohmic drop from the device. Here we can see that both Pt-WS_2_ and Ni-WS_2_ have the lowest HFR values, indicating a better electrical conductivity along the PEM cell,^47^ while the opposite was observed for Co-WS_2_ and undoped WS_2_. Interestingly, the low HFR for Ni-WS_2_ does not result in a large J^Max^, suggesting that the performance is dominated by other properties. It is also interesting to note that for both WS_2_ and Pt-WS_2_ the HFR increases as the current increases. This could be related to their morphology, where dense and less porous structures (Figure 1a–b and Figures S1–S3) affect the release of gases from the electrocatalyst/membrane interface toward the gas diffusion layer and flow field, leading to localized high-pressure points as the reaction rate increases, which might increase the interfacial contact resistance between the catalyst and membrane and in consequence the HFR. However, detailed studies are required to better understand this phenomenon.

Figure 3e shows the Nyquist plots obtained at 0.05 A cm^–2^ after the last polarization curve (Figure 3c). The series resistance (R_S_) from the EIS study (Figure S11) agrees with the HFR data (Figure 3d). For the charge transfer resistance (R_ct_), related to the overall water-splitting activity, the following trend was observed: Pt-WS_2_ < Co-WS_2_ ≈ Ni-WS_2_ < WS_2_. Figure 3f–g and Figure S12 display the current transients at 1.9 V. During both potentiostatic tests, Pt-WS_2_ and Co-WS_2_ showed good stability and progressive activation. However, Ni-WS_2_ exhibited a continuous drop in the current.

Finally, the long-term operational stability of the Pt-WS_2_ MEA was evaluated by applying a two-step galvanostatic process consisting of a current density of 0.25 A cm^–2^ for 170 h, followed by 1.0 A cm^–2^ for 380 h. This stability test was performed using a replica of the Pt-WS_2_ MEA, which exhibited identical performance to the Pt-WS_2_ MEA studied in Figure 3. The evolution of the cell voltage is shown in Figure 4a. An initial decrease of 0.06 V was observed during the first 170 h, corresponding to the low current density period. This is associated with a decrease in the HFR (Figure 4b), likely due to improved contact between interfaces. During the high current density regime (1 A cm^–2^), the potential increased to 1.92 V and remained nearly constant during the entire test, decreasing only 0.02 V by the end of the test. Meanwhile, the HFR increased only 1 mΩ when the current density was increased to 1 A cm^–2^ and remained stable for the duration of the test. These results clearly show that expanded WS_2_ containing traces of Pt is a great candidate as a cathode electrocatalyst for PEM water electrolysis.

**Figure 4:**
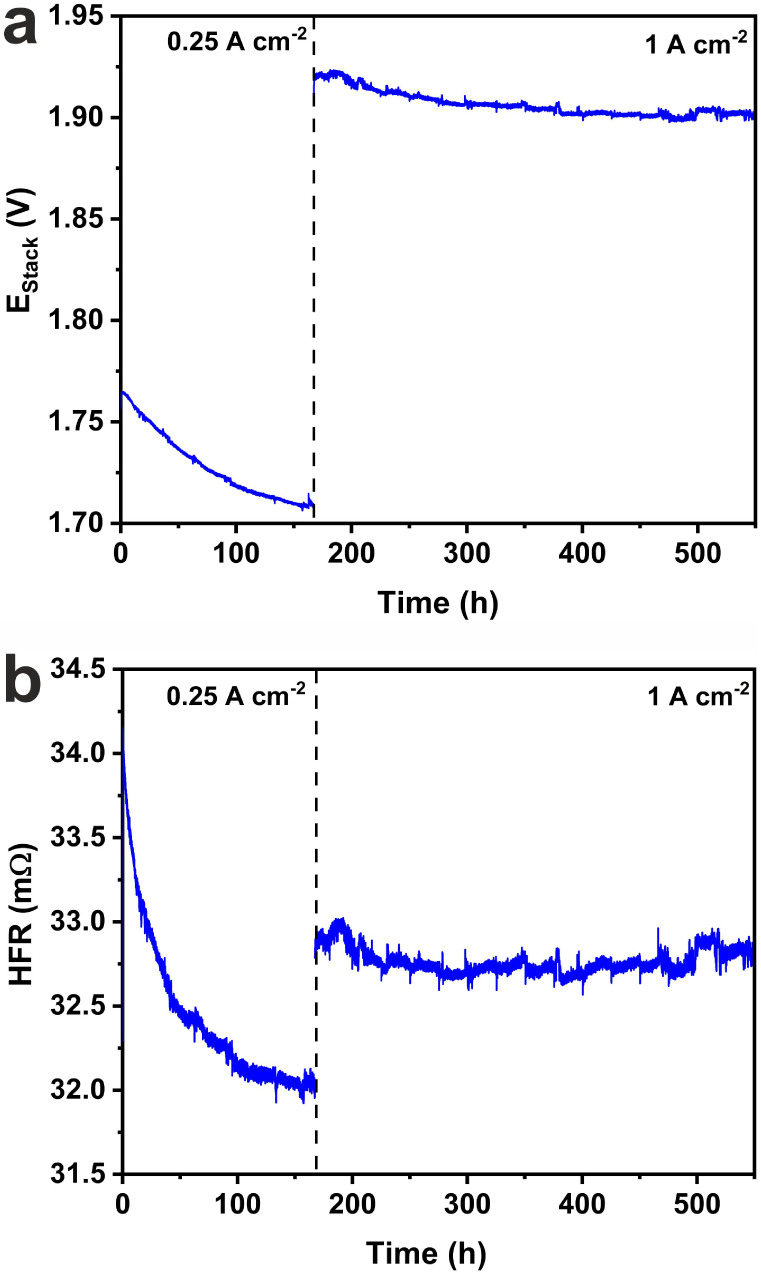
Galvanostatic stability test of the Pt-WS_2_ MEA in a PEM water electrolyzer. (a) Evolution of the cell voltage during low (0.25 A cm^–2^) and high (1 A cm^–2^) current density regimes. The entire galvanostatic test lasted for 550 h. (b) Evolution of the high-frequency resistance during the test period.

### Chemical States of TM-WS_2_

The W 4f spectrum of WS_2_ (Figure 5a) reveals three distinct pairs of peaks. The first pair at 32.1/34.3 eV (W 4f_7/2_/W 4f_5/2_) corresponds to the 1T-WS_2_ phase. The second pair at 32.8 and 35.5 eV can be assigned to the 2H-WS_2_ phase. According to the peak areas, the as-produced WS_2_ has a 1T:2H ratio of 0.9:1 (∼47% 1T-WS_2_). There is some minor content of WO_3_ as indicated by the features at 36.2/38.2 eV. The presence of both WS_2_ phases was also confirmed by the S 2p spectrum (Figure 5b), where peaks at 161.9/162.7 eV (S 2p_3/2_/S 2p_1/2_) have been assigned to the 1T-WS_2_ phase. Similarly, the 2H-WS_2_ phase was also observed by the S 2p_3/2_/S 2p_1/2_ features at higher binding energies (163.2/164.1 eV). Note that the positions of the S 2P_3/2_ and S 2p_1/2_ peaks are shifted toward higher binding energies, likely due to oxidation effects and the presence of SO_*x*_ species.^48,49^

**Figure 5:**
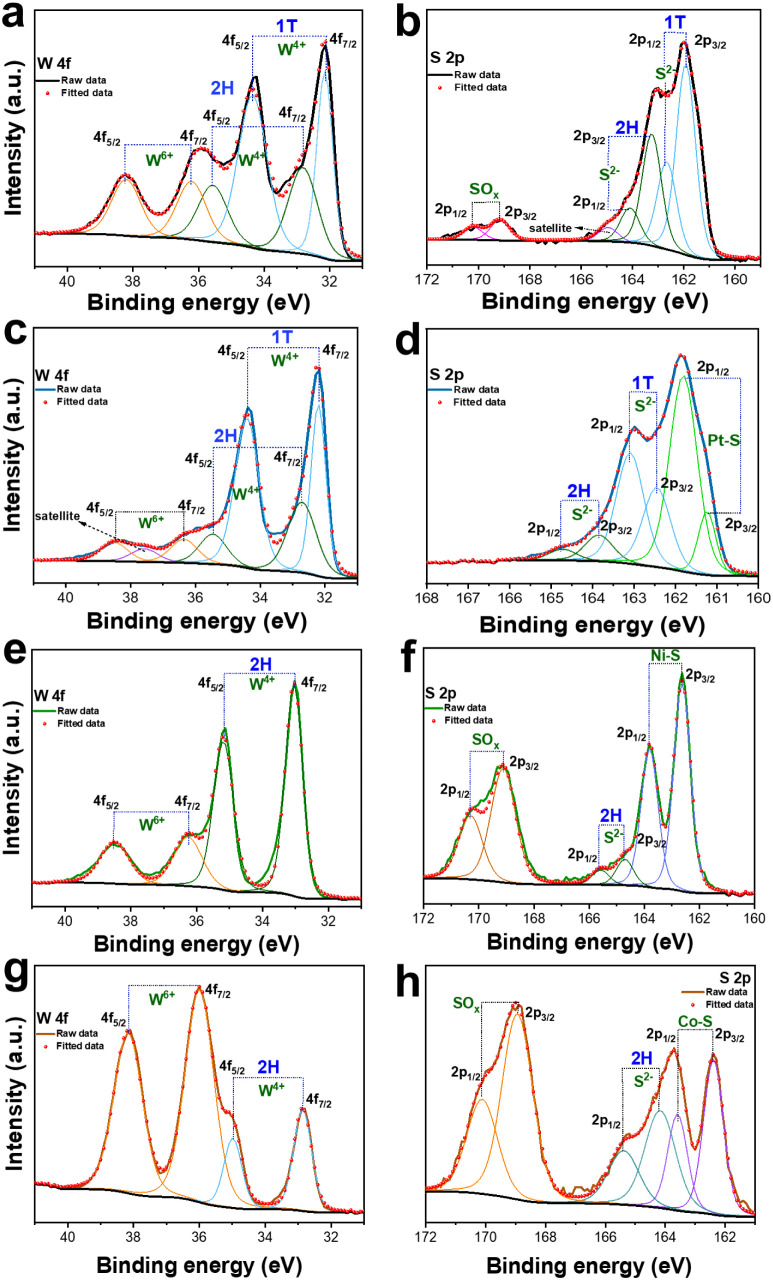
XPS core-level spectra of W 4f and S 2p: (a, b) WS_2_, (c, d) Pt-WS_2_, (e, f) Ni-WS_2_, and (g, h) Co-WS_2_.

After the incorporation of Pt, the W 4f spectrum of Pt-WS_2_ (Figure 5c) showed an increase in the area of the W 4f_7/2_/W 4f_5/2_ (32.2/34.4 eV) components (1T-WS_2_), compared to the peaks at 32.7/35.5 eV (2H phase). This change in the peak area leads to a 1T:2H phase ratio of 2.3:1 (∼70% 1T-WS_2_). Furthermore, a notable decrease in the intensity of the W^6+^ features (36.2/38.2 eV) indicates a lower contribution of WO_3_. These results suggest that the incorporation of Pt leads to charge redistribution that destabilizes the semiconducting 2H phase and facilitates the formation of the metallic 1T phase, a process seen in phase engineering through doping with transition metals.^13,37,50^ The S 2p spectrum of Pt-WS_2_ (Figure 5d) was deconvoluted into three distinct doublets located at 161.2/161.8 eV, 162.4/163.1 eV, and 163.8/164.7 eV, which are attributed to different sulfur chemical environments. The first set of peaks (161.2/161.8 eV) is assigned to sulfur bound to platinum (Pt–S). The second doublet at 162.4/163.1 eV corresponds to terminal sulfide (S^2–^) ions in the 1T phase of WS_2_. The third set (163.8/164.7 eV) is associated with S atoms in the 2H phase or bridging S^2–^ species in the WS_2_ lattice, indicating the presence of unsaturated S atoms at the metal-S sites. Notably, no peaks corresponding to sulfate groups or oxidized sulfur species were observed. Pt doping has been reported to occupy sulfur vacancies or locally compensate electronic distortions, thereby passivating reactive sites that typically lead to SO_*x*_ formation.^51,52^ The analysis of Pt 4f spectra (Figure 6a) reveals peaks at 73.5/75.5 eV (4f_7/2_/4f_5/2_) attributed to the intermediate oxidation state Pt^δ+^, while peaks at 73.8/77.0 eV are associated with Pt^2+^ species with binding energies close to those of Pt–O and Pt–S. The formation of Pt^δ+^ has been previously linked to charge transfer between Pt and S atoms.^12,42,53–55^

**Figure 6:**
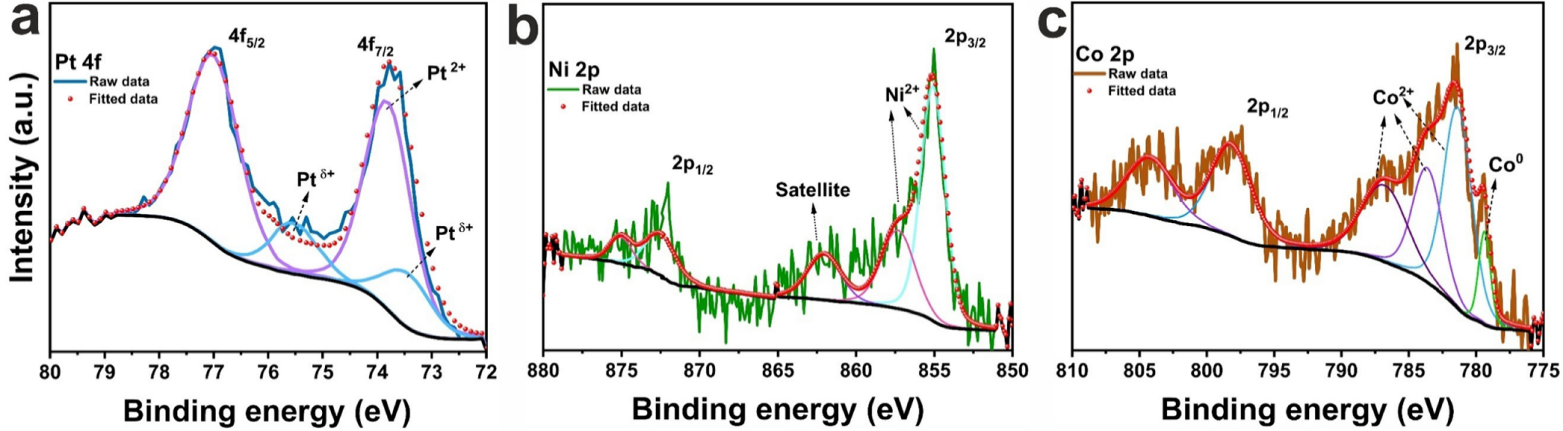
XPS spectra of (a) Pt 4f for Pt-WS_2_, (b) Ni 2p for Ni-WS_2_, and (c) Co 2p for Co-WS_2_.

On the other hand, the W 4f spectra of Ni-WS_2_ (Figure 5e) and Co-WS_2_ (Figure 5f) exhibit two sets of peaks corresponding to W^4+^ and W^6+^ species. The W^4+^ features can be assigned only to 2H-WS_2_. No peaks were observed at lower binding energies, suggesting that neither Ni nor Co promoted the formation of the metallic 1T phase. In addition, a larger content of W^6+^ was observed in both samples when compared to undoped WS_2_. Both S 2p spectra (Figure 5g–h) revealed a doublet at 162.4/163.58 eV (2p_3/2_/2p_1/2_) assigned to metal–sulfur bonds, characteristic of unsaturated sulfur atoms at transition metal sites. Another doublet at 164.1/165.39 eV (2p_3/2_/2p_1/2_) corroborates the presence of 2H-WS_2_. Additional features observed at higher binding energies, specifically the doublet at 168.5/170.1 eV (2p_3/2_/2p_1/2_), indicate the presence of a sulfate group (SO_*x*_),^37,49,56^ a feature that is not observed in Pt-WS_2_. Finally, the Ni 2p spectrum (Figure 6b) shows binding energies at 855.1/872.4 eV (2p_3/2_/2p_1/2_) for Ni^2+^.^51,57^ The Co 2p spectrum (Figure 6c) exhibits characteristics of metallic cobalt (779.4 eV) and Co^2+^ with peaks at 781.4/798.3 eV (2p_3/2_/2p_1/2_).^11,58,59^ See Table S4 for a summary of the XPS binding energies and their associated chemical states.

## Discussion

One of the main challenges with layered WS_2_ is that its most stable crystal structure, the 2H phase, is a semiconductor with poor interaction with hydrogen, resulting in low catalytic activity toward the HER. The incorporation of Pt into WS_2_ favors the formation of the metallic and highly active 1T phase of WS_2_, even at ultralow Pt concentrations. XPS studies reveal a 1T:2H ratio of 2.3:1, in contrast to undoped WS_2_ with a nearly equal contribution (1T:2H = 0.9:1). Meanwhile, the addition of Ni or Co restricted the formation of the 1T phase and led to significant tungsten and sulfur oxidation. This highlights the distinct role of Pt in promoting the phase transformation of WS_2_ toward the 1T phase. Since all materials were produced under identical conditions and only differ in the dopant metal, these results indicate that the stabilization of the 1T phase is dopant-specific rather than an intrinsic consequence of the hydrothermal synthesis method. The preferential transition to 1T-WS_2_ with Pt incorporation could be attributed to the mixed oxidation states of Pt (Pt^δ+^ and Pt^2+^), leading to strong Pt–S interaction and charge redistribution in WS_2_. Such electron redistribution is known to destabilize the trigonal coordination of the 2H phase and stabilize the octahedral coordination characteristic of the 1*T*/1T′ phases in TMDCs, similar to phase transitions promoted by lithiation of TMDCs.^13,42,60–63^

Importantly, the absence of any detectable contribution of Pt^0^ in the Pt 4f XPS spectra, together with the ultralow Pt content (<0.3 at%), rules out metallic Pt clusters as the dominant catalytic material. In addition, Pt-WS_2_ exhibits minimal contribution of W^6+^ and negligible SO_*x*_ features, suggesting the formation of unsaturated Pt–S–W centers, analogous to those reported for Pt-MoS_2_ systems.^52,64^ These active sites are characterized by nearly an optimal hydrogen energy. Moreover, the presence of Pt can result in enhanced electronic states near the Fermi level, benefiting the electrical conductivity and charge transfer processes.^65,66^ Overall, the addition of just 0.3 at% of Pt led to a reduction of 41% in η_10_, a 48% smaller Tafel slope, a change in the rate-determining step, 78% smaller charge transfer resistance, and 14 times larger surface area when compared to undoped WS_2_. All these indicate that trace amounts of Pt promote the formation of the 1T phase and passivate structural defects that are prone to oxidation and expose catalytically active surfaces.

Under PEMWE operating conditions, most electrocatalysts showed an increase in performance after the 70 h testing protocol. Pt-WS_2_ achieved a current density of 1.0 A cm^–2^@1.92 V and 1.75 A cm^–2^@2.1 V. The current density at 2.1 V is 270% larger than that of pristine WS_2_ (0.64 A cm^–2^@2.1 V) and 225% better than Ni-WS_2_ (0.78 A cm^–2^@2.1 V) and Co-WS_2_ (0.78 A cm^–2^@2.1 V). We also observed that the trend for the water-splitting onset potential (Pt-WS_2_ (1.37 V) < WS_2_ ≈ Co-WS_2_ (1.39 V) < Ni-WS_2_ (1.48 V)) does not directly correlate with the performance at high current densities. Therefore, other factors such as interface characteristics, diffusion of reactants, and gas buildup might play a key role under such conditions. In addition, the Pt-WS_2_ MEA exhibits an excellent operational stability of more than 550 h when operated at high current density. Finally, we also observed that Ni-WS_2_ had a lower high-frequency resistance in PEMWE cells, suggesting a better conductivity throughout the MEA assembly. However, it does not translate into better catalytic performance, likely due to the lower content of 1T-WS_2_ and partial oxidation of sulfur species. But this brings up an interesting path: nickel could be used in combination with platinum, producing a cathode with a large content of 1T-WS_2_, better transport characteristics, and lower interfacial resistance, ultimately improving the conductivity of the MEA assembly.

## Conclusions

We produced expanded multilayer WS_2_ containing a mixture of 2*H*/1T phases. The addition of low amounts of Pt, Ni, and Co allows tuning of the crystal structure and oxidation state of chemical species. While the addition of Ni and Co improves the HER performance, it is not enough to make the material a compelling catalyst. However, the addition of trace amounts of Pt significantly benefits the stabilization of metallic 1T-WS_2_ and suppresses the formation of oxidized surface species. These effects are observed despite the Pt content being well below the threshold to form metallic Pt clusters, indicating that the observed catalytic activity mostly originates from doping effects and the presence of the 1T-WS_2_ rather than direct Pt catalysis. The resulting changes lead to better electronic conductivity, enhanced hydrogen adsorption energies, larger electrochemically active surface area, and faster HER kinetics, all while maintaining excellent operational stability. Furthermore, we demonstrated that the benefits seen in the three-electrode system can be transferred to a complete PEMWE cell, where the Pt-WS_2_ cathode continued to demonstrate great performance and stability for more than 550 h of operation at 1 A cm^–2^. Although the activity of Pt-WS_2_ does not surpass that of commercial Pt/C cathodes, this study shows that WS_2_ is a viable cathode electrode for PEMWE systems when combined with trace levels of noble metals. Overall, our study is a step forward in reducing the content of PGMs to below the technical target stated by the US Department of Energy of 0.5 mg cm^–2^.^67^

## Supplementary Material



## Notes

Electron microscopy images, EDX elemental mapping, Raman spectra, DFT activity maps, capacitance measurements, EIS fitting, and potentiostatic curves of all materials; PEM galvanostatic curves of the reference sample (PDF)
