# Supplementary material for: Ultralow Platinum Content in Defect-Rich Tungsten Disulfide: Approaching Platinum Performance in Proton Exchange Membrane Water Electrolyzers
Source: ACS Appl Mater Interfaces. 2026 Apr 13;18(16):23300–11. doi: 10.1021/acsami.6c03423 (PMC13133780; doi:10.1021/acsami.6c03423)
Supplement: Supplementary file 1 [file am6c03423_si_001.pdf]

Supporting Information

Ultra-low Platinum Content in Defect-rich Tungsten Disulphide: Approaching Platinum Performance in Proton Exchange Membrane Water Electrolysers

Elena Puentes-Prado,[1] Esdras J. Canto-Aguilar,[1] Alice Kuzhikandathil,[1] Mouna Rafei,[1] Tugce Ustunel,[1,2] and Eduardo Gracia-Espino.[1,*]

[1] *Department of Physics, Umeå University, SE-901 87 Umeå, Sweden.*

[2] *Permascand AB, Folketshusvägen 50, SE-841 99 Ljungaverk, Sweden.*

*Corresponding author: Eduardo Gracia-Espino (eduardo.gracia@umu.se

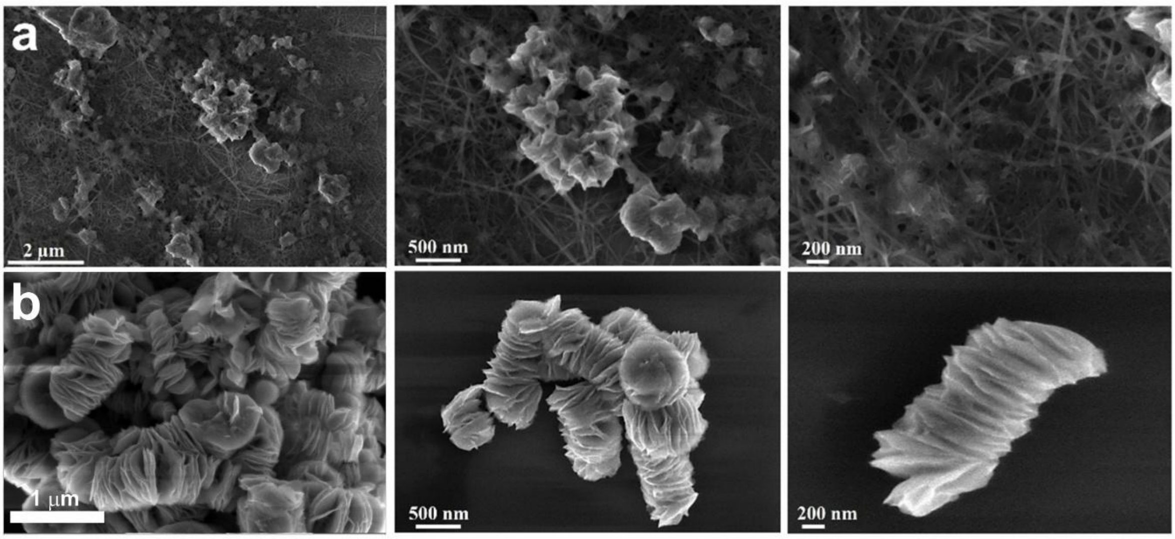

Figure S1. *SEM images of (a) WS_2 and (b) Pt-WS_2.*

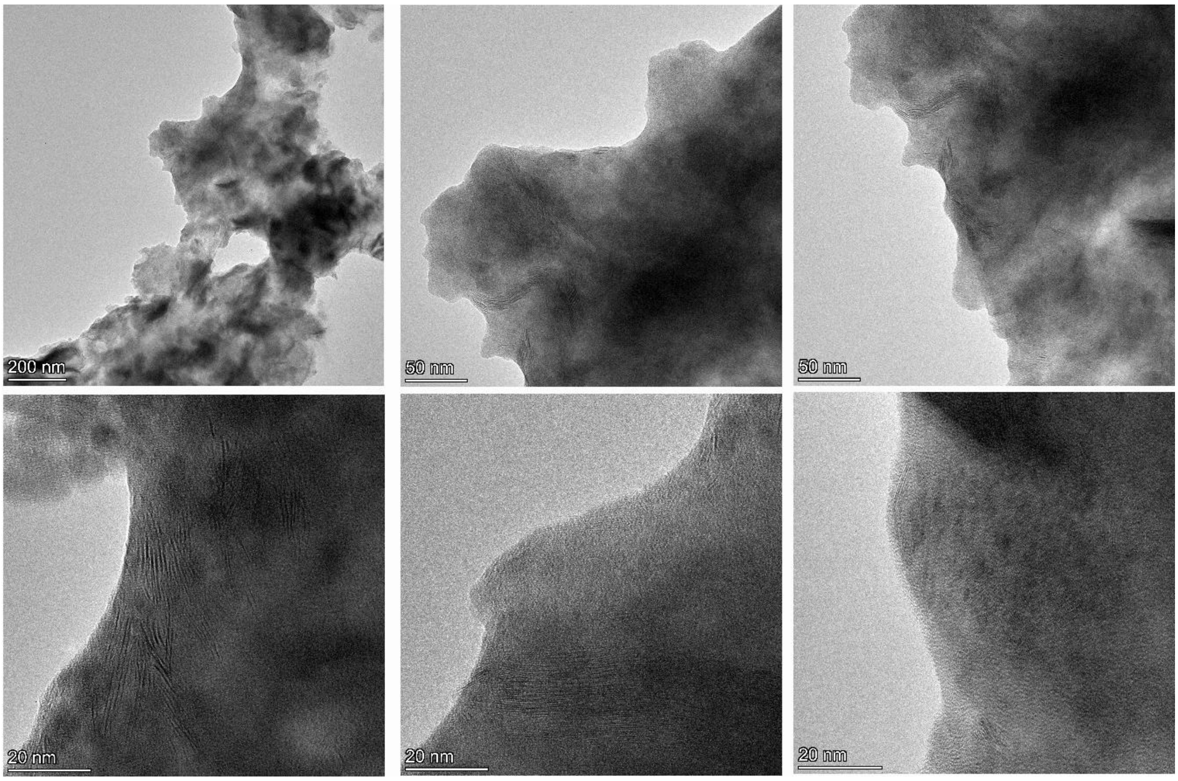

Figure S2. *High-resolution TEM micrographs of WS_2.*

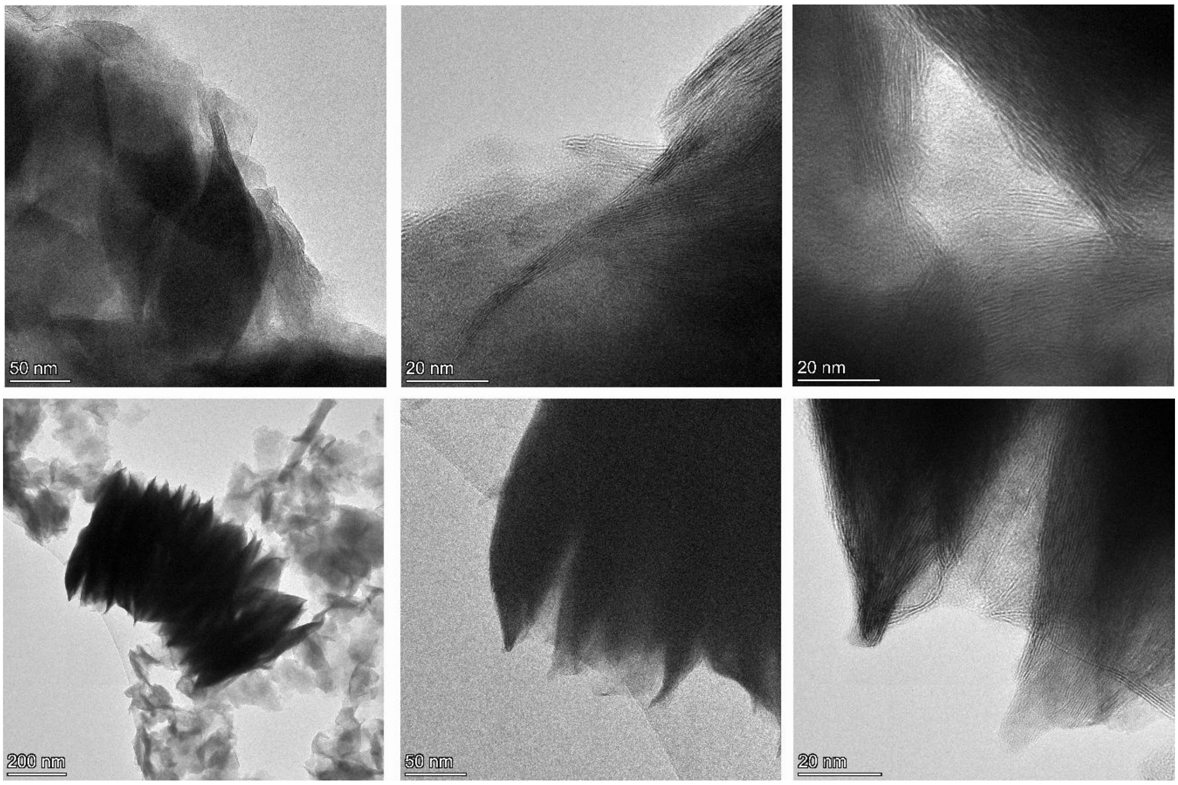

Figure S3. *High-resolution TEM micrographs of Pt-WS_2.*

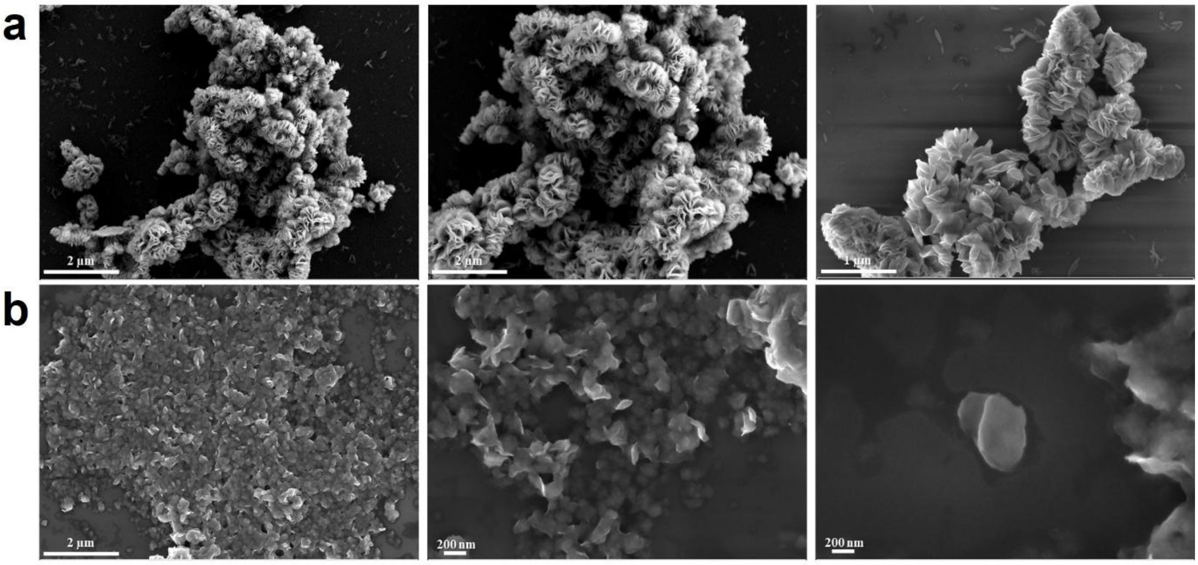

Figure S4. *SEM images of (a) Ni-WS_2, and (b) Co-WS_2.*

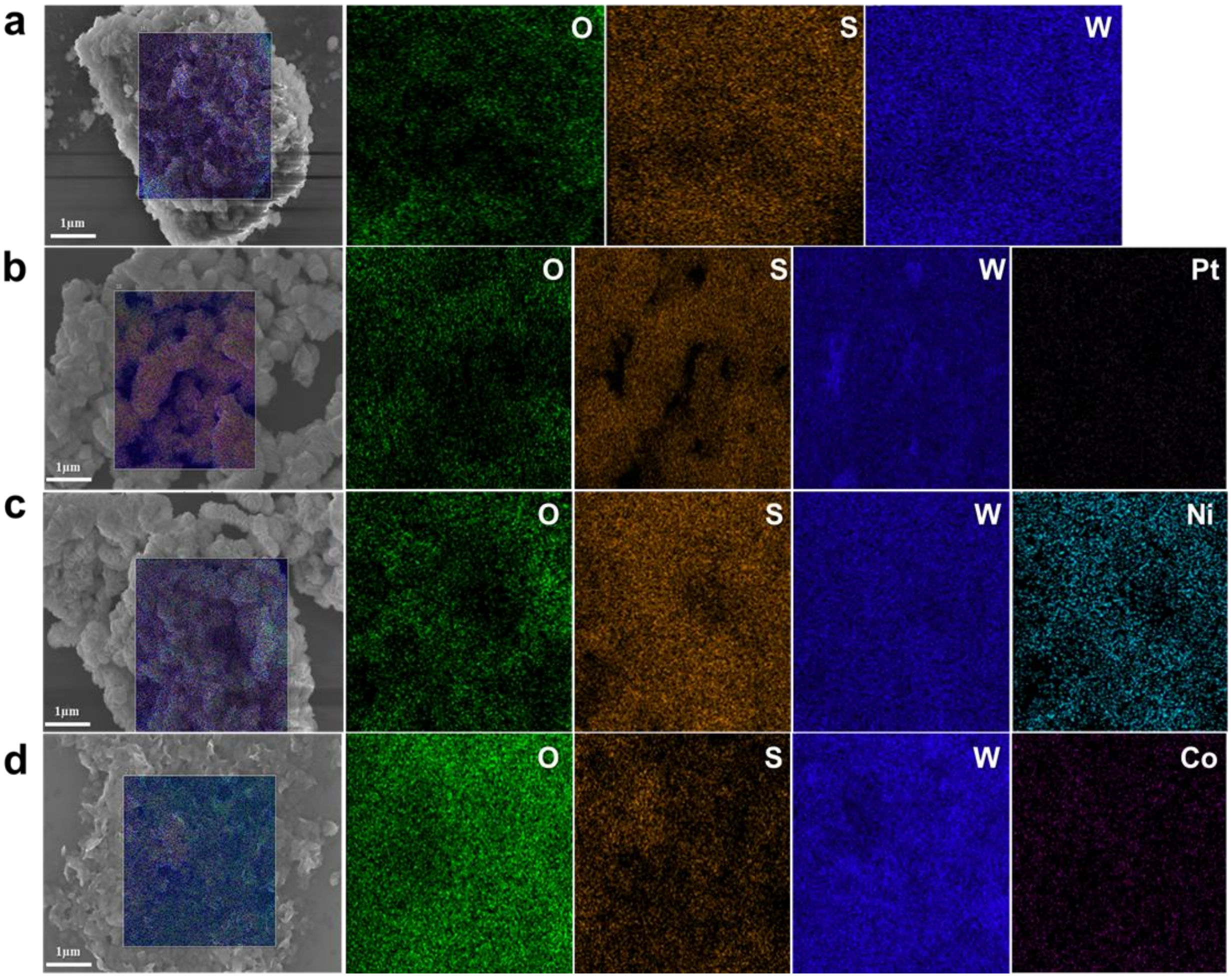

Figure S5. *EDX elemental mapping of (a) WS_2, (b) Pt-WS_2, (c) Ni-WS_2, and (d) Co-WS_2.*

Table S1. *Elemental composition obtained from EDX analysis. The composition is an average of two measurements performed on different spots.*

**Sample**	**W (At%)**	**S (At%)**	**TM (At%)**	**W:S (At)**	**W:TM**
**WS_2**	26.7	43.0	-	1 : 1.61	-
**WS_2–Pt**	33.1	52.3	<0.1	1 : 1.58	331:1
**WS_2–Ni**	28.9	52.2	1.8	1 : 1.81	16:1
**WS_2–Co**	16.0	11.1	0.9	1 : 0.69	18:1

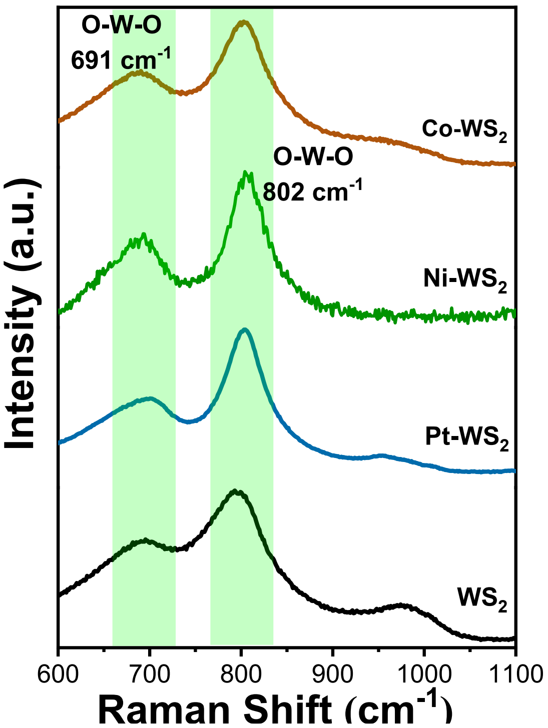

Figure S6. *Raman spectra of WS_2, Pt-WS_2, Ni-WS_2, Co-WS_2 in the 600-1100 cm^{-1} region. This is the complementary Raman shown in Figure 1d in the main manuscript.*

Table S2. *Raman shifts and full width at half maximum (FWHM) of peaks shown in Figure 1d in the main manuscript.*

Sample	Raman Shift (cm^{-1})			FWHM (cm^{-1})		
	LA(M)	E^1_{2g}	A_{1g}	*LA(M)*	E^1_{2g}	A_{1g}
WS_2	170.6	350.8	415.8	10.2	11.5	10.8
Pt-WS_2	-	348.1	413.9	-	6.8	8.4
Ni-WS_2	171.0	348.0	412.9	9.7	10.6	8.84
Co-WS_2	170.8	349.0	414.4	9.9	11.1	9.1

Table S3. *Electrochemical HER performance. Overpotential (η_{10}) needed to reach a -10 mA cm^{-2}. Charge transfer resistance (R_{CT}) and ohmic resistance (R_S) obtained from EIS studies. ECSA obtained from measurements of the capacitance of the double-layer.*

Catalyst	η_{10} (mV)	Tafel slope (mV dec^{-1})	R_{ct} from EIS at -0.2 V vs RHE (Ωcm^{-2})	R_s (Ωcm^{-2})	ECSA ($cm^{-2}g^{-1}$)
**WS_2**	614	207	327.2	2.7	9.9
**Pt-WS_2**	360	107	75.4	2.2	139.0
**Ni-WS_2**	537	180	80.7	3.1	30.7
**Co-WS_2**	568	205	101.7	7.3	12.8

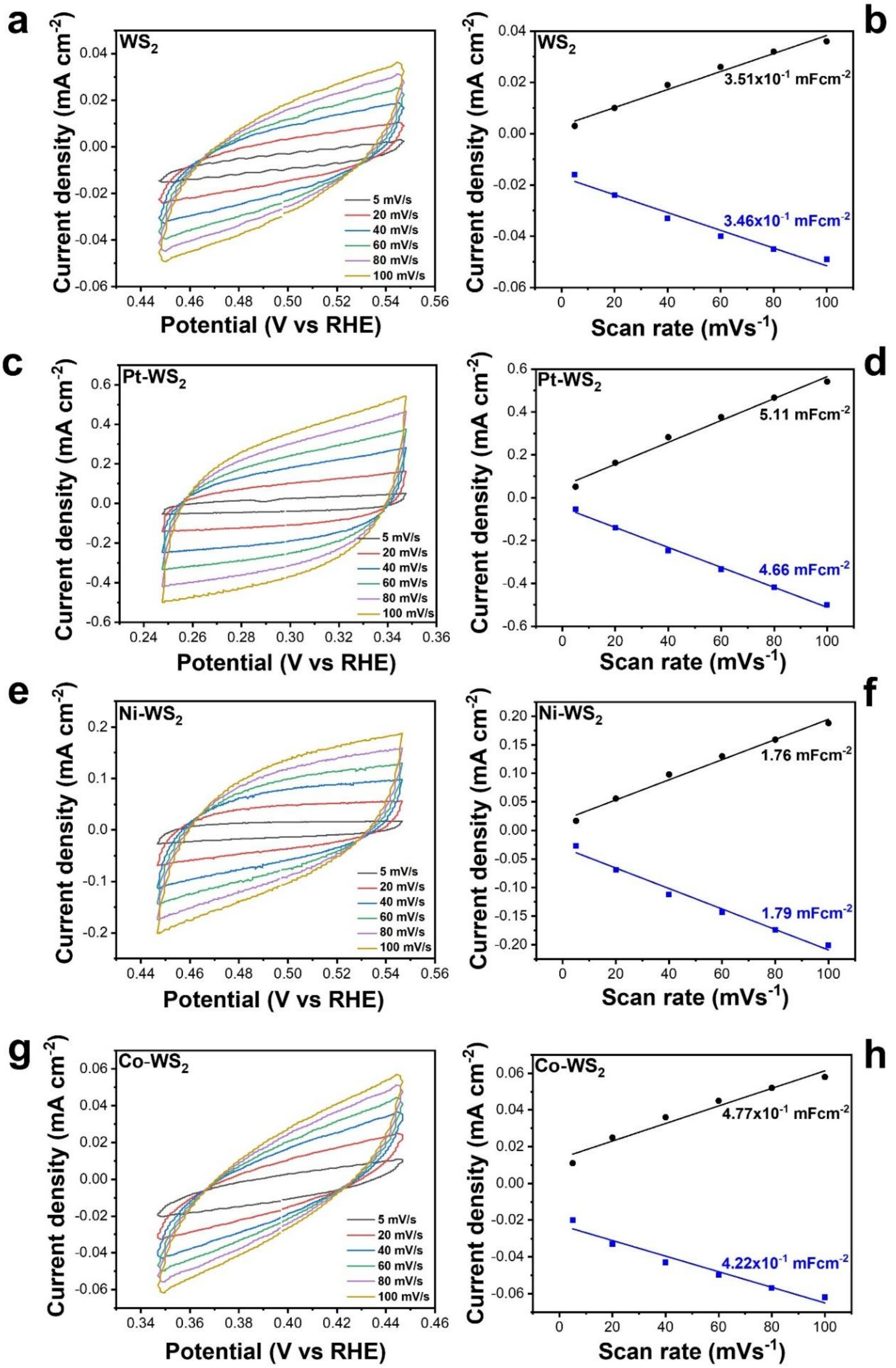

Figure S7*. CV curves and C_{dl} values of (a, b) WS_2, (c, d) Pt- WS_2, (e, f) Ni-WS_2, and (g, h) Co-WS_2.*

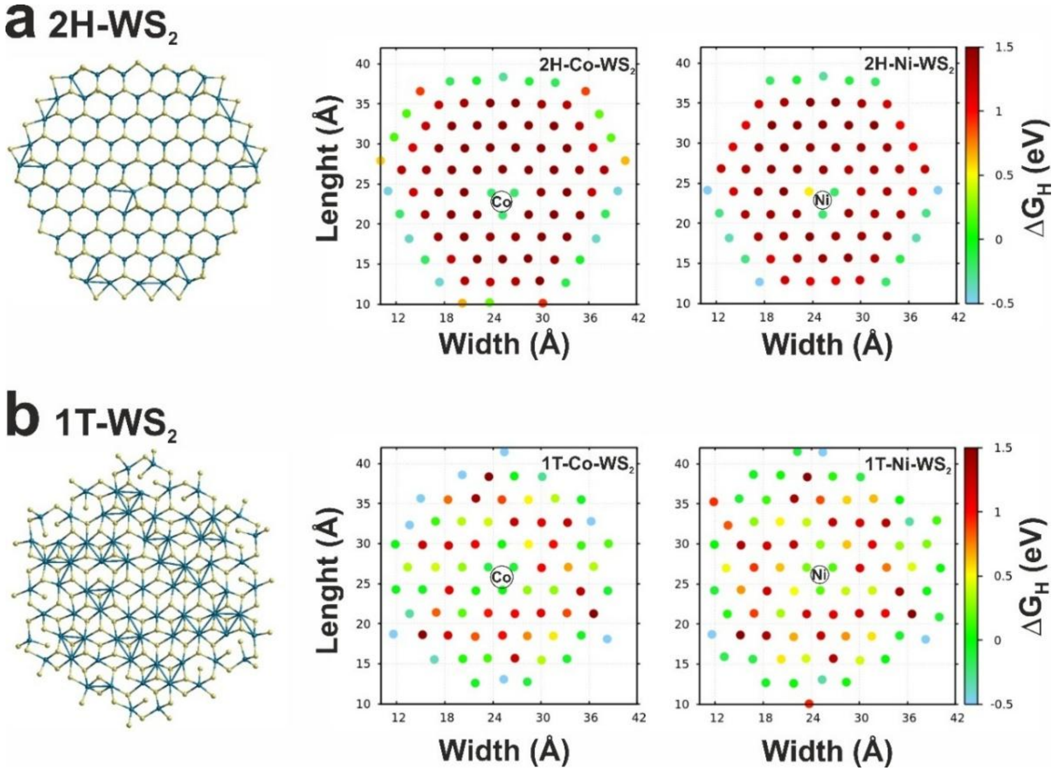

Figure S8. *HER activity maps of Co-WS$_2$ and Ni-WS$_2$. WS$_2$ doped systems with (a) 2H and (b) 1T phase. The atomic model used to evaluate the hydrogen adsorption energy is shown. Sulphur atoms are represented by yellow spheres, while tungsten with blue. The position of the Co atom (or Ni atom) is indicated with a black circle. Positive (negative) values of hydrogen adsorption free energy (ΔG_H) indicate a weak (strong) interaction with hydrogen. Sites with optimal hydrogen interaction ($\Delta G_H \approx 0$) are green.*

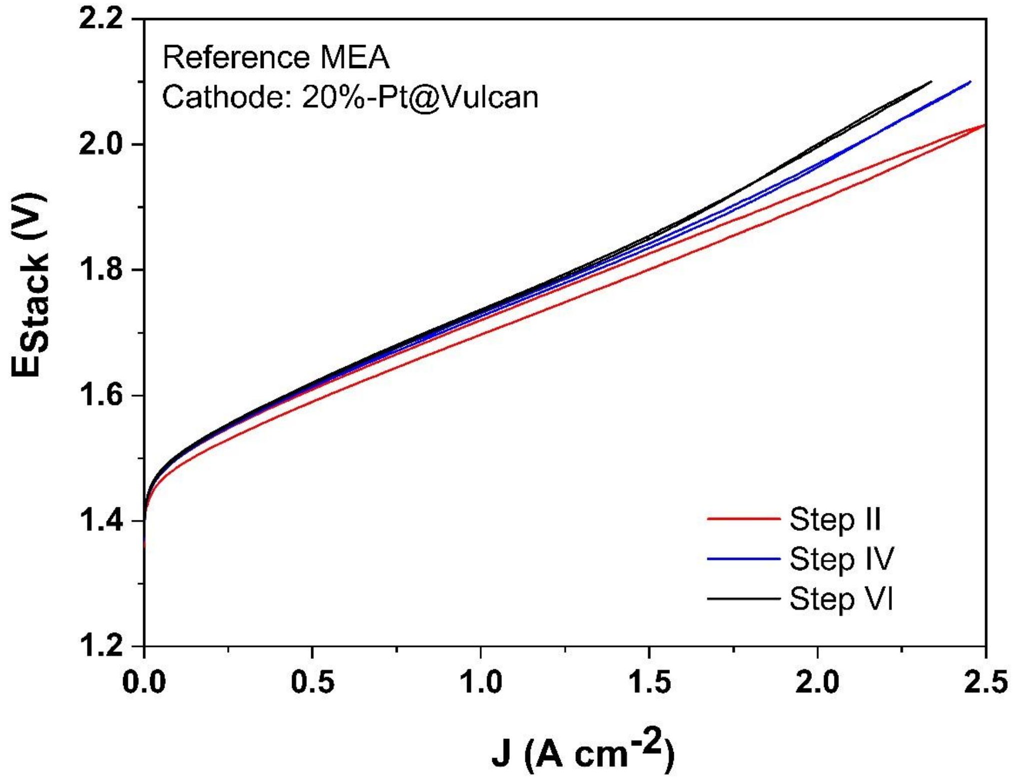

Figure S9. *Polarisation curve of the reference MEA containing 20%-Pt@Vulcan as cathode. The polarisation curves were performed in step, II, IV, and VI from the testing protocol depicted in Figure 3a in the main manuscript.*

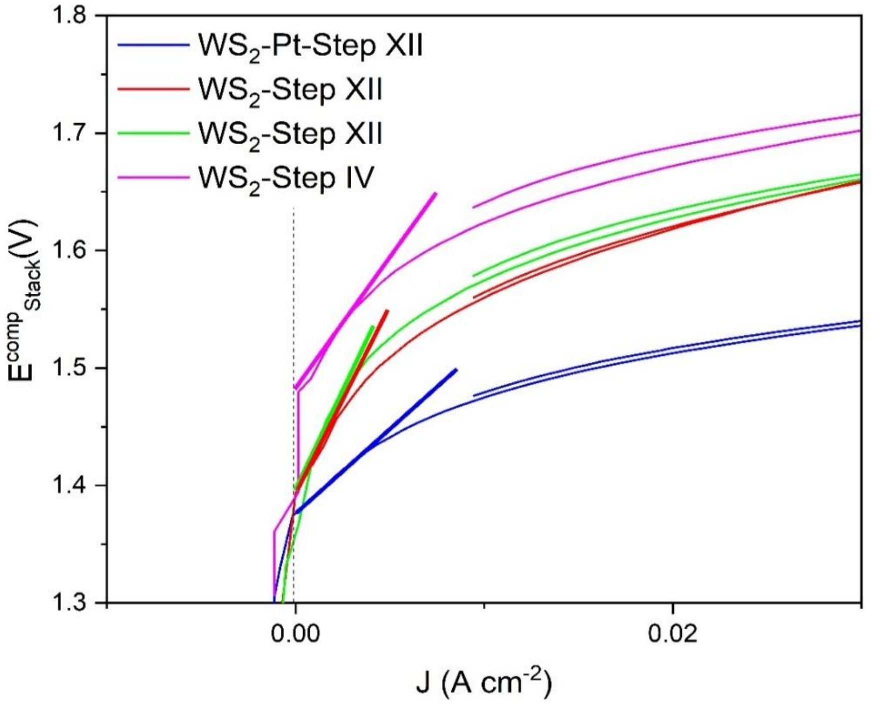

Figure S10. *Water splitting onset potential calculated for the galvanostatic polarisation curves in Figure 2a in the main manuscript.*

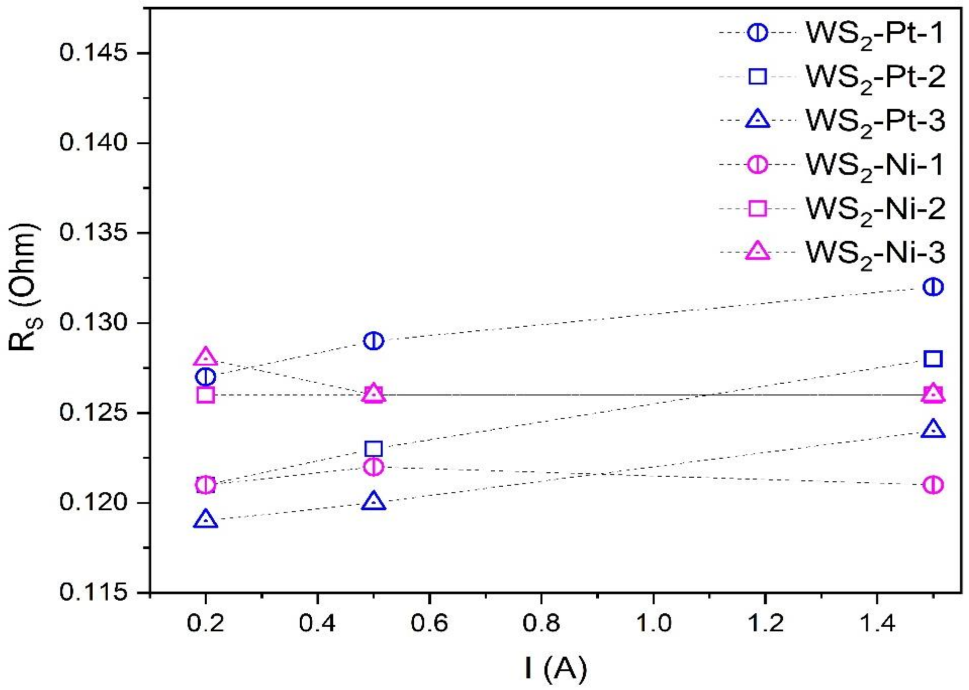

Figure S11. *Series resistance (R_S) as a function of the current applied, calculated from the EIS spectra obtained in steps III, V, and VI for Pt-WS_2 and Ni-WS_2 materials.*

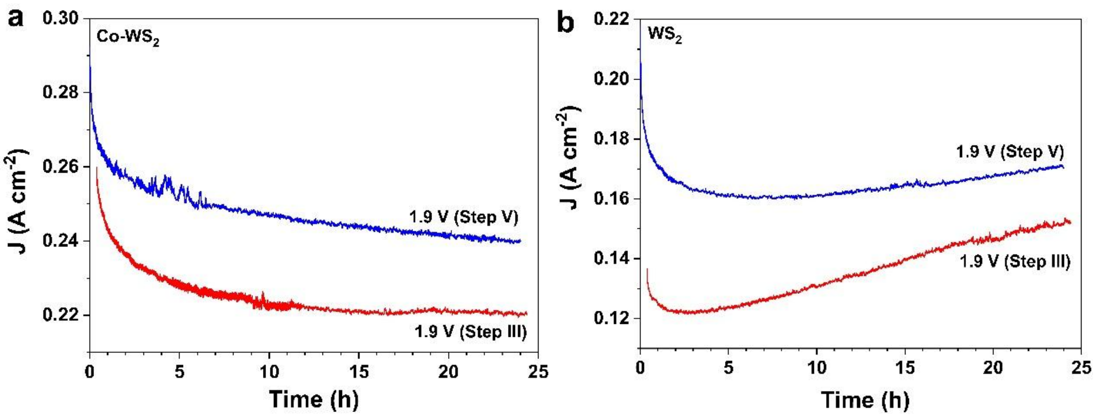

Figure S12. *Current transients for (a) Co-WS_2, and (b) WS_2 of both potentiostatic studies performed in step III and V of the test protocol. See Figure 3a in the main manuscript.*

Table S4. *Peak positions and FWHM values obtained from high-resolution XPS peak deconvolution of WS_2 and TM-WS_2.*

<table>
<tr><th colspan="2">Assignment</th><th>Orbital</th><th>Binding energy (eV)</th><th>FWHM (eV)</th></tr>
<tr><td rowspan="6">WS_2</td><td rowspan="2">1T</td><td>$4f_{7/2}$</td><td>32.1</td><td rowspan="2">1.3</td></tr>
<tr><td>$4f_{5/2}$</td><td>34.3</td></tr>
<tr><td rowspan="2">2H</td><td>$4f_{7/2}$</td><td>32.8</td><td rowspan="2">1.2</td></tr>
<tr><td>$4f_{5/2}$</td><td>35.5</td></tr>
<tr><td rowspan="2">W-O</td><td>$4f_{7/2}$</td><td>36.2</td><td rowspan="2">1.4</td></tr>
<tr><td>$4f_{5/2}$</td><td>38.2</td></tr>
<tr><td rowspan="9">Pt-WS_2</td><td rowspan="2">1T</td><td>$4f_{7/2}$</td><td>32.2</td><td rowspan="2">1.5</td></tr>
<tr><td>$4f_{5/2}$</td><td>34.4</td></tr>
<tr><td rowspan="2">2H</td><td>$4f_{7/2}$</td><td>32.7</td><td rowspan="2">1.3</td></tr>
<tr><td>$4f_{5/2}$</td><td>35.5</td></tr>
<tr><td rowspan="2">W-O</td><td>$4f_{7/2}$</td><td>36.4</td><td rowspan="2">1.0</td></tr>
<tr><td>$4f_{5/2}$</td><td>38.5</td></tr>
<tr><td>Pt^{2+}</td><td>$4f_{7/2}$</td><td>73.8</td><td>1.1</td></tr>
<tr><td rowspan="2">$Pt^{\delta+}$</td><td>$4f_{7/2}$</td><td>73.5</td><td rowspan="2">1.3</td></tr>
<tr><td>$4f_{5/2}$</td><td>75.5</td></tr>
<tr><td rowspan="2">Ni-WS_2</td><td rowspan="2">Ni^{2+}</td><td>$2p_{3/2}$</td><td>855.1</td><td rowspan="2">1.5</td></tr>
<tr><td>$2p_{1/2}$</td><td>872.4</td></tr>
<tr><td rowspan="3">Co-WS_2</td><td>Co^0</td><td>$2p_{3/2}$</td><td>779.4</td><td>1.0</td></tr>
<tr><td rowspan="2">Co^{2+}</td><td>$2p_{3/2}$</td><td>781.4</td><td rowspan="2">~1.5</td></tr>
<tr><td>$2p_{1/2}$</td><td>798.3</td></tr>
</table>
